# Association between change in handgrip strength and cognitive function in Korean adults: a longitudinal panel study

**DOI:** 10.1186/s12877-021-02610-2

**Published:** 2021-12-01

**Authors:** Hyunkyu Kim, Seung Hoon Kim, Wonjeong Jeong, Sung-In Jang, Eun-Cheol Park, Youseok Kim

**Affiliations:** 1 Department of Preventive Medicine, Yonsei University College of Medicine, Seoul, Republic of Korea; 2 Institute of Health Services Research, Yonsei University, Seoul, Republic of Korea; 3 Department of Psychiatry, Yonsei University College of Medicine, Seoul, Republic of Korea; 4 Department of Public Health, Graduate School, Yonsei University, Seoul, Republic of Korea; 5 Department of Hospital administration, Graduate School of Public Health, Yonsei University, 50-1 Yonsei-ro, Seodaemun-gu, Seoul, 03722 Republic of Korea

**Keywords:** Handgrip strength, Cognitive impairment, Cognitive function, Aging, Korean longitudinal study of aging

## Abstract

**Background:**

Muscular function, such as handgrip strength, has been suggested as an associated factor for cognitive impairment. This study investigated the association between temporal change in handgrip strength and cognitive function using longitudinal, nationwide data from Korean older adults.

**Methods:**

Our study used data from the Korean Longitudinal Study of Aging (KLoSA). The analysis covered 6696 participants who had taken the handgrip strength test and Mini-Mental State Examination (MMSE) from 2006 to 2018. We adopted general estimating equations to assess the temporal effect of handgrip strength change on cognitive function.

**Results:**

After adjusting for covariates, we observed an association between handgrip strength and low MMSE scores (β = − 0.3142 in men, β = − 0.2685 in women). Handgrip strength as a continuous variable was positively correlated with MMSE scores after adjustment (β = 0.0293 in men, β = 0.0347 in women). The group with decreased handgrip strength over time also showed greater odds for mild cognitive impairment (OR = 1.23, 95%CI = 1.05–1.27 in men, OR = 1.15, 95%CI = 1.05–1.27 in women) and dementia (OR = 1.393, 95%CI = 1.18–1.65 in men, OR = 1.19, 95%CI = 1.08–1.32 in women).

**Conclusions:**

This study identified the relationship between handgrip strength change and cognitive function among South Korean adults. According to our large, longitudinal sample, decreasing handgrip strength was associated with decline in cognitive function.

**Supplementary Information:**

The online version contains supplementary material available at 10.1186/s12877-021-02610-2.

## Background

As aging progresses, impairment in cognitive function may arise, and its intensified form, dementia, is considered a major health problem worldwide [1]. Cognitive impairment induces socio-economic burden by causing poor quality of life, hospitalization, increased mortality, and poverty [2–5]. However, cognitive decline generally occurs gradually. Its onset or early phase is not easy to detect, with patients remaining undiagnosed until they display some functional impairment. Progression is likewise difficult to stop because cognitive impairment is a degenerative disease. Therefore, many studies have focused on methods to prevent cognitive impairment, including early detection and intervention, for example, by prescribing drugs such as donepezil, which is now widely used in clinical settings [6, 7].

Studies have suggested the various factors associated with cognitive impairment and the ways to prevent decline based on those factors. The patient’s neuropsychiatric condition, including depression, insomnia, or drug use [8–13], or chronic disease, such as hypertension or diabetes [14, 15], have been associated with cognitive decline. Other studies have pointed to several modifiable health behaviors including exercise, and body conditions, such as body mass index (BMI) and muscle mass, as being associated with dementia [16]. Among these, the relationship between handgrip strength, which can be easily measured, and cognitive impairment is becoming increasingly apparent [17].

A longitudinal study conducted in the United States positively linked handgrip strength with cognitive function, and a cohort study on Mexican Americans showed the association between baseline handgrip strength and Mini-Mental State Examination (MMSE) scores [18, 19]. A recent study using a longitudinal panel also showed that lower handgrip strength was associated with a higher odds ratio for cognitive impairment in aging Americans [20]. Similarly, a longitudinal study conducted in South Korea indicated the association between greater handgrip strength and a lower odds ratio of cognitive impairment [13]. However, few studies have investigated the association between changes in handgrip strength and cognitive impairment [17]. The confirmation of such an association in a large sample and through longitudinal design study would be useful as a basis for preventing cognitive decline by modulating handgrip strength via strength exercises. Therefore, we aimed to investigate the association between changes in handgrip strength and cognitive function in the Korean adult population based on a panel study, after adjusting for covariates that were assumed to affect cognitive function.

## Methods

### Study population and data

The data analyzed in this study were taken from the Korean Longitudinal Study of Aging database (KLoSA). The KLoSA is a longitudinal panel survey of nationally representative samples of community-dwelling adults aged above 45 years, and has been conducted every 2 years since 2006 [21]. The baseline data, gathered in 2006, include 10,254 Korean adults who have been interviewed by trained interviewers. The survey gathers information on respondents’ family background, demographics, family composition, health, employment, income, assets, subjective expectations, and subjective quality of life. The seventh wave of KLoSA, conducted in 2018, covered an effective sample of 6136 from the original panels and 804 newly included panels. In the present study, we employed survey data from 2006 to 2018, for a total of seven datasets. After deleting data with missing values for variables, we analyzed data from 6696 participants, including 2999 men and 3697 women. For statistical analysis, each change in handgrip strength from 2006 to 2018 was treated as an individual case rather than the population number itself. As KLoSA provides data in de-identified form which is open data for academic use, the need for informed consent was waived by the Institutional Review Board of Yonsei University’s Health System (4–2021-0307).

### Measures

#### Mini-mental state examination

To measure the cognitive function of the participants, we referred to their MMSE scores. The MMSE is a widely used tool for measuring cognitive function and screening for cognitive impairment in older adults [22, 23]. The validity of the Korean version of the MMSE (k-MMSE) has been established for its usefulness in screening for cognitive impairment [24]. With a total score of 30, the MMSE’s cut-off level for mild cognitive impairment is 23 and that for dementia is 19 [25]. We used total scores for analysis to reveal detailed results regarding the association.

#### Handgrip strength

The KLoSA measures handgrip strength in kilograms using a handgrip dynamometer (Hand Grip Meter 6103, Tanita, Tokyo, Japan). Participants are asked to squeeze the dynamometer twice for each hand, and the mean value among four trials is recorded. Our data analysis excluded participants who declined to perform the test owing to physical problems. To analyze the association between changes in handgrip strength and cognitive function, we calculated the differences in reported values per wave. Handgrip strength was considered as both a categorized and a continuous variable in the analysis. As handgrip strength has been found to be significantly different between men and women in previous studies, we analyzed the data with stratification by sex [26, 27]. We also standardized the handgrip strength change by calculating the percentage change in handgrip strength from the handgrip strength of the original wave. The asymmetry in handgrip strength was calculated by subtracting the lower value from the higher value of handgrip strength. The change of the asymmetry per wave was then divided into two groups: 1) decreased, 2) same or increased.

#### Covariates

We considered demographic and health-related factors as covariates in the analysis. Demographic characteristics included age, educational level, residential region, working status, household income, participation in social activities, and number of cohabiting generations. Health-related factors included smoking/alcohol use status, number of chronic medical conditions, BMI, and perceived health status. All the multivariable models controlled for all covariates unless stated otherwise.

### Statistical analysis

All statistical analyses were performed separately for men and women to rule out the effect of sex in terms of the difference in handgrip strength on cognitive function. We employed analysis of variance to investigate and compare the general characteristics of the study population. We also constructed a generalized estimating equation model for regression analysis between MMSE scores and change in handgrip and other covariates. The analysis was conducted twice using the different variable types of change in handgrip strength: the two categorical groups of change in handgrip strength and the continuous variable of the same. The results were presented as regression coefficients (β) and 95% confidence intervals (95%CI). We performed subgroup analyses for a detailed study of the interaction between change in handgrip strength and other variables associated with MMSE scores. All analyses were carried out using SAS Version 9.4 (SAS Institute, Cary, North Carolina, USA). The results were considered statistically significant at *p* < 0.05.

## Results

Table 1 gives the baseline characteristics of the study population stratified by sex. The unadjusted comparison showed no statistical difference in MMSE scores between the two groups of handgrip strength change in both sexes. Other covariates, such as age, educational level, region of residence, working status, household income, participation in social activities, BMI, and perceived health status, showed significant differences in MMSE scores for both sexes. The comparison of mean MMSE scores between two groups in Wave 2 and Wave 7 are presented in Supplementary Table 1. Both groups showed no statistically significant differences in mean MMSE scores for both Waves 2 and 7.

**Table 1:** Baseline characteristics of the study population according to Mini-Mental State Examination scores

	Men (***N*** = 2999)					Women (***N*** = 3697)				
	Participants		MMSE			Participants		MMSE		
	N	%	Mean ± S.D.		***p***-value	N	%	Mean ± S.D.		***p***-value
**Change in handgrip strength**					0.6874					0.2907
Same or increased	1196	39.9	26.891	3.397		1673	45.3	25.385	4.798	
Decreased	1803	60.1	26.846	3.654		2024	54.7	25.253	4.813	
**Age (years)**					< 0.0001					< 0.0001
45–54	831	27.7	28.331	2.030		1131	30.6	27.842	2.396	
55–64	938	31.3	27.527	2.689		1084	29.3	26.522	3.404	
65–74	874	29.1	26.122	3.492		991	26.8	23.784	4.798	
≥75	356	11.9	23.517	5.396		491	13.3	19.904	6.070	
**Education level**					< 0.0001					< 0.0001
Elementary school or less	908	30.3	24.816	4.463		2016	54.5	23.321	5.293	
Middle school	522	17.4	27.080	2.905		635	17.2	27.047	2.981	
High school	1066	35.5	27.804	2.619		879	23.8	28.023	2.290	
University or beyond	503	16.8	28.346	2.095		167	4.5	28.503	1.951	
**Region**					< 0.0001					< 0.0001
Metropolitan	1240	41.3	27.432	3.045		1580	42.7	26.157	4.196	
Small or medium cities	991	33.0	26.898	3.499		1206	32.6	25.080	5.055	
Rural	768	25.6	25.904	4.138		911	24.6	24.157	5.176	
**Working status**					< 0.0001					0.0198
Working	1905	63.5	27.569	2.685		1225	33.1	26.581	3.541	
Non-working	1094	36.5	25.637	4.438		2472	66.9	24.684	5.211	
**Household income**					< 0.0001					0.0097
Quartile 1 (low)	600	20.0	24.760	4.534		991	26.8	23.188	5.256	
Quartile 2	816	27.2	26.799	3.210		987	26.7	25.401	4.440	
Quartile 3	846	28.2	27.508	2.959		907	24.5	26.114	4.484	
Quartile 4 (high)	737	24.6	27.910	2.837		812	22.0	26.905	4.043	
**Participation in social activities**					< 0.0001					< 0.0001
No	528	17.6	25.011	4.919		819	22.2	22.933	5.708	
Yes	2471	82.4	27.260	3.045		2878	77.8	25.990	4.283	
**Smoking**					0.0327					0.7566
Current	1179	39.3	27.160	3.145		107	2.9	24.019	4.939	
Former	778	25.9	26.719	3.533		38	1.0	24.395	5.274	
Never	1042	34.7	26.638	3.960		3552	96.1	25.362	4.792	
**Alcohol Intake**					0.1605					0.0340
Yes	1895	63.2	27.104	3.255		709	19.2	26.616	3.625	
No	1104	36.8	26.452	3.982		2988	80.8	25.004	4.997	
**Number of chronic medical conditions**					0.1283					0.5450
None	1539	51.3	27.402	3.153		1672	45.2	26.53	4.14	
1	895	29.8	26.535	3.730		1134	30.7	24.85	4.83	
≥2	565	18.8	25.920	4.010		891	24.1	23.62	5.31	
**Number of cohabiting generations**					0.0212					0.0541
Couple	1394	46.5	26.305	3.859		1783	48.2	24.812	4.731	
Two generations	1266	42.2	27.582	2.855		1415	38.3	26.339	4.443	
Over two generations	339	11.3	26.484	4.091		499	13.5	24.196	5.492	
**BMI**					0.0180					< 0.0001
Underweight	89	3.0	24.944	4.971		114	3.1	23.360	6.044	
Normal weight	1321	44.0	26.513	3.782		1628	44.0	25.076	5.105	
Overweight	1012	33.7	27.292	3.290		1024	27.7	25.974	4.278	
Obesity	544	18.1	27.270	2.917		818	22.1	25.571	4.389	
Severe obesity	33	1.1	26.273	3.785		113	3.1	22.850	4.881	
**Perceived health status**					< 0.0001					< 0.0001
Healthy	1752	58.4	27.744	2.675		1673	45.3	26.993	3.531	
Average	855	28.5	26.035	3.803		1222	33.1	24.634	4.809	
Unhealthy	392	13.1	24.742	4.866		802	21.7	22.843	5.710	

*BMI* body mass index, Underweight: BMI < 18.5, Normal weight: 18.5 ≤ BMI < 23, Overweight: 23 ≤ BMI < 25, Obesity: 25 ≤ BMI < 30, Severe obesity: 30 ≤ BMI

Table 2 shows the multiple regression analysis results for associations between MMSE scores and handgrip strength change groups after adjusting for covariates. Compared with the same or increased handgrip strength group, the decreased handgrip strength group showed highly significant regression coefficients (− 0.3142 in men and − 0.2685 in women). Decreased handgrip strength was associated with decreased MMSE scores in both sexes. The results of other covariates are also shown in Table 2. Higher age showed a significant association with decreased MMSE scores, albeit with smaller regression coefficients. The decreased handgrip strength group also showed statistically significant odds ratios for mild cognitive impairment (OR = 1.23 in men, OR = 1.15 in women) and dementia (OR = 1.39 in men, OR = 1.19 in women), as illustrated in Table 3.

**Table 2:** Results of the GEE analysis of handgrip strength change in two groups and Mini-Mental State Examination scores

	Men		Women	
	β	95%CI	β	95%CI
**Changes in Handgrip strength**				
Same or Increased	Ref.		Ref.	
Decreased	−0.3142	(− 0.4129 -0.2154)	− 0.2685	(− 0.3732 -0.1637)
**Age**				
45–54	Ref.		Ref.	
55–64	− 0.1218	(− 0.2560 0.0124)	− 0.1374	(− 0.2760 0.0012)
65–74	− 0.5936	(− 0.7915 -0.3957)	−1.2507	(− 1.4799 -1.0214)
≥75	− 1.6447	(− 1.9444 -1.3449)	−3.4250	(−3.7685 -3.0814)
**Education level**				
Elementary school or less	−2.2240	(−2.4879 -1.9601)	−2.9756	(−3.2934 -2.6577)
Middle school	− 0.8777	(− 1.1216 -0.6337)	−1.0498	(− 1.3567 -0.7428)
High school	−0.5350	(− 0.7142 -0.3557)	−0.5839	(− 0.8543 -0.3136)
University or beyond	Ref.		Ref.	
**Region**				
Metropolitan	Ref.		Ref.	
Small or Medium Cities	−0.3659	(− 0.5544 -0.1774)	−0.5500	(− 0.7737 -0.3262)
Rural	−0.3564	(− 0.5755 -0.1373)	−0.8134	(− 1.0690 -0.5578)
**Working status**				
Working	Ref.		Ref.	
Non-working	− 0.4528	(− 0.6053 -0.3003)	−0.4327	(− 0.5817 -0.2836)
**Household income**				
Quartile 1 (low)	−0.5991	(− 0.8564 -0.3417)	−0.3133	(− 0.5657 -0.0609)
Quartile 2	−0.0653	(− 0.2554 0.1249)	0.0357	(−0.1743 0.2458)
Quartile 3	−0.0110	(− 0.1703 0.1484)	−0.0498	(− 0.2293 0.1296)
Quartile 4 (high)	Ref.		Ref.	
**Participation in social activities**				
No	−0.9662	(− 1.1680 -0.7644)	−1.2188	(− 1.4130 -1.0247)
Yes	Ref.		Ref.	
**Smoking**				
Current	0.2185	(−0.1002 0.3107)	0.2520	(− 0.3558 1.0495)
Former	0.1053	(0.0150 0.4221)	0.3468	(−0.3385 0.8425)
Never	Ref.		Ref.	
**Alcohol Intake**				
Yes	0.0944	(− 0.0633 0.2521)	0.3999	(0.2034 0.5964)
No	Ref.		Ref.	
**Number of chronic medical conditions**				
None	Ref.		Ref.	
1	−0.0207	(− 0.1878 0.1464)	0.0023	(−0.1920 0.1966)
≥2	− 0.1014	(−0.3174 0.1145)	−0.2783	(− 0.5405 -0.0161)
**Number of cohabiting generations**				
Couple	0.4467	(0.1589 0.7346)	0.7265	(0.4234 1.0296)
Two generation	0.3116	(0.0398 0.5835)	0.4248	(0.1206 0.7291)
Over two generation	Ref.		Ref.	
**BMI**				
Underweight	0.2462	(−0.2392 0.7316)	−0.3675	(− 0.8535 0.1185)
Normal weight	Ref.		Ref.	
Overweight	0.1452	(−0.0042 0.2945)	0.3958	(0.2294 0.5622)
Obesity	0.1092	(−0.0672 0.2856)	0.5409	(0.3336 0.7483)
Severe obesity	0.2504	(−0.4315 0.9323)	−0.2268	(− 0.7595 0.3060)
**Perceived health status**				
Healthy	Ref.		Ref.	
Average	−0.2811	(− 0.4087 -0.1536)	−0.3218	(− 0.4640 -0.1796)
Unhealthy	−1.2703	(− 1.5197 -1.0208)	−1.1481	(− 1.3674 -0.9288)

*BMI* body mass index, Underweight: BMI < 18.5, Normal weight: 18.5 ≤ BMI < 23, Overweight: 23 ≤ BMI < 25, Obesity: 25 ≤ BMI < 30, Severe obesity: 30 ≤ BMI

**Table 3:** Results of the GEE analysis of handgrip strength change and mild cognitive impairment/dementia

	Mild Cognitive Impairment (20 ≤ MMSE < 24)				Dementia (MMSE < 20)			
	**Men**		**Women**		**Men**		**Women**	
	**Adjusted OR**	**95%CI**	**Adjusted OR**	**95%CI**	**Adjusted OR**	**95%CI**	**Adjusted OR**	**95%CI**
**Changes in Handgrip strength**								
Same or Increased	Ref.				Ref.			
Decreased	1.23	(1.07–1.41)	1.15	(1.05–1.27)	1.39	(1.18–1.65)	1.19	(1.08–1.32)

*All variables in Table 2 were included in the GEE model

Table 4 shows the results of the multiple regression analysis between MMSE scores and handgrip strength change in continuous values with the same covariates in Table 2. The regression coefficients were 0.0293 in men and 0.0347 in women, indicating a high level of statistical significance. The results affirm the positive association between change in handgrip strength and MMSE total scores in both sexes. The decrease in handgrip strength asymmetry was associated with lower MMSE scores (β = − 0.1476 in men and β = − 0.1755 in women) as shown in Table 5.

**Table 4:** Results of the GEE analysis of handgrip strength change in continuous variables and Mini-Mental State Examination scores

	Men		Women	
	β	95%CI	β	95%CI
**Handgrip strength change (kg)**	0.0293	(0.0210 0.0375)	0.0347	(0.0232 0.0463)
**Hangrip strength change (%)**	0.0077	(0.0052 0.0101)	0.0049	(0.0030 0.0068)

*All variables in Table 2 were included in the GEE model. Handgrip strength change were shown in absolute value (kg) and percent change from previous handgrip strength (%).*All variables in Table 2 were included in the GEE model. Handgrip strength change were shown in absolute value (kg) and percent change from previous handgrip strength (%)

**Table 5:** Results of the GEE analysis of handgrip strength asymmetry change and Mini-Mental State Examination scores

	Men		Women	
	β	95%CI	β	95%CI
**Handgrip strength asymmetry change (kg)**				
Same or Increased	Ref.		Ref.	
Decreased	−0.1476	(− 0.2485 -0.0466)	−0.1755	(− 0.2822 -0.0688)

*All variables in Table 2 were included in the GEE model. Handgrip strength asymmetry was calculated by subtracting lower handgrip strength from higher handgrip strength. The change of handgrip strength asymmetry between 2 years were grouped into Same/Increased and Decreased

## Discussion

In this study, we identified that change in handgrip strength is associated with cognitive function in community-dwelling South Korean adults. The group with decreased handgrip strength was associated with low cognitive function when compared with the group with the same or increased handgrip strength. Furthermore, the value of the handgrip strength change was positively correlated with the MMSE scores in the study population.

Our results were generally consistent with previous studies on the relationship between change in handgrip strength and cognitive function in different populations. Previous studies showed that low baseline handgrip strength was associated with cognitive decline [28]. Christensen et al. reported that handgrip strength change, rather than initial strength, predicts changes in memory task performance [29]. MacDonald et al. suggested that biological changes, including grip strength change, share significant time-varying associations with change in cognitive function; handgrip strength decline is associated with cognitive function decline [29]. Several studies reported that physical frailty, including grip strength, was associated with cognition, suggesting that they might share common pathology [30, 31]. Compared with previous works, our results newly established that handgrip strength change is associated with cognitive function in older adults.

In our study, a decreased handgrip strength asymmetry was associated with a lower MMSE score, as shown in Table 5. Mcgrath et al. showed that handgrip strength asymmetry was associated with lower cognitive functioning [32]. The change in handgrip strength through aging or exercise could be different, resulting in a stronger or a weaker hand; thus, the change in asymmetry showed different patterns related to the asymmetry itself, with respect to association with cognitive function.

The etiology of the association between change in handgrip strength and cognitive function has not been established, although several possible explanations have been suggested. One is that physical activity increases the size of the prefrontal and hippocampal brain areas, thereby reducing cognitive decline [33–35]. Changes in handgrip strength can reflect the changes in the physical activity of individuals; thus, decreased handgrip might reflect reduced cognitive function. The frailty concept could be one explanation of the relationship between handgrip and reduced cognitive function. Handgrip strength decline could be an early and readily detectible indicator of frailty, which includes consequent decline of cognitive function in older adults. Previous studies showed that physical frailty, including being underweight, having weaker grip strength, and having a poor performance on the chair stand test was associated with cognitive decline [36, 37]. Another explanation is that cognitive function and handgrip strength might share a common domain of the brain, such as the frontal executive function area; decreased handgrip strength and cognitive decline might occur simultaneously.

Meanwhile, decreased cognitive function might also induce the change in handgrip strength. A study on the direction of the relationship between strength and cognitive function showed a significant bi-directional relationship [13]. Thus, muscular strength and cognitive function might share common causes of change. In our study, we could not exclude the possibility that participants with low cognitive function might have had difficulty in maintaining physical activity, including strength exercises, which could lead to the decrease in their handgrip strength. Further research should be performed to test the directionality or causality of the two variables.

This study has several limitations. First, as the data were collected via a survey, the results might be biased. Second, we excluded the data of those who did not answer the important covariate questions, which may have induced the underestimation of cognitive decline in the participants. Third, we could not include biological risk factors, which could have led us to overlook some important confounding variables. Several biological factors have been established as risk factors of cognitive impairment in adults, and future studies should include these in regression model analyses [38]. Fourth, as we used brief measurements for cognitive function, the impact of handgrip strength changes on different neurocognitive domains could not be analyzed in this study. Previous studies showed that grip strength had different effects on cognitive domains [39, 40]. Further research using a comprehensive neurocognitive test would refine our study results. Finally, cause and effect could not be established because our study did not use a prospective design, which could be used to assess the causality of change in handgrip strength vis-à-vis change in cognitive function.

Nonetheless, the strengths of our study include the relatively large sample size and longitudinal design. Our results can be representative of the Korean adult population. Another strength is that this study used standardized tools to measure muscle strength and cognitive function; therefore, the results are readily applicable for further study. Moreover, given our use of the change in handgrip strength rather than baseline strength, the present results can be referenced when introducing lifestyle modifications, such as strength exercises, for older adults to help them maintain or increase their handgrip strength, which can prevent cognitive function decline.

In conclusion, this study identified the relationship between changes in handgrip strength and cognitive function among South Korean adults. Decreased handgrip strength was associated with cognitive decline in our longitudinal, large-sample study. Further studies exploring the underlying mechanisms of the association between handgrip strength and cognitive impairment, as well as the preventive effect of increasing the former, could provide valuable strategies for treating and preventing cognitive impairment in clinical settings.

## Supplementary Information

**Additional file 1:.**

## Data Availability

The datasets analyzed during the current study are available in the KLoSA repository, https://survey.keis.or.kr/eng/klosa/databoard/List.jsp
