# Supplementary material for: Association between change in handgrip strength and cognitive function in Korean adults: a longitudinal panel study
Source: BMC Geriatr. 2021 Dec 1;21:671. doi: 10.1186/s12877-021-02610-2 (PMC8638365; doi:10.1186/s12877-021-02610-2)
Supplement: Supplementary file 1 — Additional file 1:. [file 12877_2021_2610_MOESM1_ESM.docx]

| **Supplementary Table 1. MMSE scores of Same or Increased and Decreased handgrip strength group in Wave 2 and Wave 7** | | | | | | | | | | | | | | | | |
| --- | --- | --- | --- | --- | --- | --- | --- | --- | --- | --- | --- | --- | --- | --- | --- | --- |
|  | **Wave 2** | | | | | | | | **Wave 7** | | | | | | | |
|  | **Male** | | | | **Female** | | | | **Male** | | | | **Female** | | | |
|  | **N(%)** | **Mean** | **SD** | **p-value** | **N(%)** | **Mean** | **SD** | **p-value** | **N(%)** | **Mean** | **SD** | **p-value** | **N(%)** | **Mean** | **SD** | **p-value** |
| **Changes in Handgrip strength** |  |  |  | 0.6874 |  |  |  | 0.2907 |  |  |  | 0.9396 |  |  |  | 0.5394 |
| Same or Increased | 1196(39.9) | 26.891 | 3.397 |  | 1763(46.6) | 25.385 | 4.798 |  | 995(41.7) | 26.747 | 3.910 |  | 1349(44.4) | 25.643 | 5.087 |  |
| Decreased | 1803(60.1) | 26.846 | 3.654 |  | 2024(53.4) | 25.253 | 4.813 |  | 1390(58.3) | 26.760 | 4.269 |  | 1686(55.6) | 25.530 | 5.065 |  |
